# Concurrent Brain Subregion Microgliosis in an HLA-II Mouse Model of Group A Streptococcal Skin Infection

**DOI:** 10.3390/microorganisms11092356

**Published:** 2023-09-20

**Authors:** Suba Nookala, Santhosh Mukundan, Bryon Grove, Colin Combs

**Affiliations:** Department of Biomedical Sciences, School of Medicine and Health Sciences, University of North Dakota, Grand Forks, ND 58202, USA; santhosh.mukundan@und.edu (S.M.); bryon.grove@und.edu (B.G.); colin.combs@und.edu (C.C.)

**Keywords:** Group A Streptococcus, HLA-II, microgliosis

## Abstract

The broad range of clinical manifestations and life-threatening infections caused by the Gram-positive bacterium, *Streptococcus pyogenes* or Group A Streptococcus (GAS), remains a significant concern to public health, with a subset of individuals developing neurological complications. Here, we examined the concurrent neuroimmune effects of subcutaneous GAS infections in an HLA-Class II (HLA) transgenic mouse model of subcutaneous GAS infection. To investigate changes in the skin–brain axis, HLA-DQ8 (DQA1*0301/DQB1*0302) mice (DQ8) were randomly divided into three groups: uninfected controls (No Inf), GAS infected and untreated (No Tx), and GAS infected with a resolution by clindamycin (CLN) treatment (CLN Tx) (10 mg/kg/5 days) and were monitored for 16 days post-infection. While the skin GAS burden was significantly reduced by CLN, the cortical and hippocampal GAS burden in the male DQ8 mice was not significantly reduced with CLN. Immunoreactivity to anti-GAS antibody revealed the presence of GAS bacteria in the vicinity of the neuronal nucleus in the neocortex of both No Tx and CLN Tx male DQ8 mice. GAS infection-mediated cortical cytokine changes were modest; however, compared to No Inf or No Tx groups, a significant increase in IL-2, IL-13, IL-22, and IL-10 levels was observed in CLN Tx females despite the lack of GAS burden. Western blot analysis of cortical and hippocampal homogenates showed significantly higher ionized calcium-binding adaptor-1 (Iba-1, microglia marker) protein levels in No Tx females and males and CLN Tx males compared to the No Inf group. Immunohistochemical analysis showed that Iba-1 immunoreactivity in the hippocampal CA3 and CA1 subregions was significantly higher in the CLN Tx males compared to the No Tx group. Our data support the possibility that the subcutaneous GAS infection communicates to the brain and is characterized by intraneuronal GAS sequestration, brain cytokine changes, Iba-1 protein levels, and concurrent CA3 and CA1 subregion-specific microgliosis, even without bacteremia.

## 1. Introduction

β-hemolytic Group A Streptococcus (*Streptococcus pyogenes*, GAS) is an important human pathogen that causes a broad spectrum of clinical presentations ranging from mild self-limiting pharyngitis and skin infections such as cellulitis and impetigo to life-threatening and often fatal invasive infections including necrotizing fasciitis (NF, aka “flesh-eating disease”), a subset of necrotizing soft tissue infections (NSTI) [1]. NF manifests as fascial necrosis and is usually accompanied by systemic signs of streptococcal toxic shock syndrome (STSS), leading to a cytokine storm and increasing the risk for multiple organ failure and mortality in susceptible individuals [1,2,3,4]. Significant inter-individual variations exist in the clinical presentation, disease severity, and outcomes of invasive GAS infections [5,6]. These depend on the heterogeneity in the virulence potential among the GAS isolates and the host risk factors, specifically variations in HLA Class II (HLA-II) alleles that determine the type of immune response elicited [7,8,9]. Despite prompt interventions, recurrent GAS infections are not uncommon. GAS mimics of host proteins can prime individuals with HLA-II predilection for increased risk of severity, outcomes, and post-streptococcal sequelae, which include neurological manifestations and neuropsychiatric disorders like Sydenham chorea and the Pediatric Autoimmune Neuropsychiatric Disorders Associated with Streptococcal infections (PANDAS) [10] subgroup of tic disorders and attention disorders [11,12,13], and risk for seizures in some cases [14]. While GAS meningitis [15] and brain abscesses have been associated with significant morbidity and mortality [16,17], the mechanisms underlying GAS neuropathogenesis remain incompletely understood. As the frontline mediators of innate and adaptive immune signaling within the brain, the brain resident immune cells astrocytes and microglia are poised to play central roles in determining infection outcomes [18,19]. Elucidating the responses of astrocytes and microglia and the localized brain cytokines they govern is crucial for understanding host defense strategies, pathological processes, and antibiotic therapy-mediated sequelae during GAS infections.

HLA-II-expressing humanized mouse models are well-established and frequently used as clinical translation models for studying mechanisms underlying dysregulated host immune responses to infections [10,20,21], streptococcal and staphylococcal superantigen responses, dermatitis, collagen-induced arthritis [22,23,24], food allergens [25], dust allergens [26], progression of demyelination and neurological deficits in Theiler’s murine encephalomyelitis virus infection [27], and brain pathology in experimental autoimmune encephalomyelitis [28,29]. Building upon our previous work [30], and given the potential for the differential presentation of immune responses and outcomes by DR and DQ alleles, in the present study, we utilized the HLA-DQ8 (DQA1*0301/DQB1*0302) mice (DQ8) to investigate the skin–brain axis and effects of clindamycin (CLN) following subcutaneous GAS infection. Our findings offer unique insights into brain cytokine changes, GAS dissemination, and subregion-specific Iba-1 immunoreactivity that endured beyond an apparent CLN-mediated resolution of skin infection.

## 2. Materials and Methods

### 2.1. Mice

20–24 weeks-old females and males expressing the human HLA-DQ8 (DQA1*0301/DQB1*0302) allele and deficient in endogenous class-II molecules [31] were used as sublethal mouse models of subcutaneous GAS infection. The genotype of the mice was confirmed by PCR-based genotyping using allele-specific oligonucleotide primers Sense 5′-AGG ATT TGG TGT ACC AGT TTA AGG GCA T-3′ and Antisense 5′-TGC AAG GTC GTG CGG AGC TCC AA-3′. The surface expression of the HLA-DQ allele was confirmed by flow cytometry (BD Biosciences, Franklin Lakes, NJ, USA) after surface staining whole blood with phycoerythrin (PE) labeled anti-HLA-DQ (clone DQ1) (BioLegend, San Diego, CA, USA) antibody [9].

### 2.2. GAS Bacteria and In Vivo Infection

We used a well-characterized and representative M1T1 GAS clinical isolate 5448 [32] for subcutaneous infections as described [9,33,34]. GAS inoculum for in vivo infections was prepared as previously reported [9,34]. Briefly, GAS 5448 was grown under static conditions for 16 h at 37 °C in a THY medium (BD Bacto (Cat# 249240) containing 1.5% (*w*/*v*) Bacto yeast extract (Cat# 212750) (Thermo Fisher Scientific, Waltham, MA, USA). The bacteria were harvested by centrifugation (610× *g*, 10 min at room temperature) and washed three times in sterile Dulbecco’s phosphate-buffered saline without calcium and magnesium (DPBS, Ca^++^/Mg^++^ free, low endotoxin, PBS^−/−^) (Thermo Fisher Scientific). The washed pellets were re-suspended in PBS^-/-^ and diluted to the desired optical density at 600 nm (OD_600_ adjusted to yield ~1 − 5 × 10^8^ CFU/0.1 mL). Actual inocula were determined by plating on trypticase soy agar containing 5% sheep blood (Thermo Fisher Scientific, Waltham, MA, USA).

In vivo infections were performed as described [9,34]. One day before infection, mice were anesthetized, and hair on the dorsal side was depilated using Nair cream (Church and Dwight Co., Inc., Lakewood, NJ, USA). Mice were randomly divided into three groups: (Group 1: No Infection (No Inf), Group 2: GAS Infected Untreated (No Tx), and Group 3: GAS Infected and clindamycin treated (CLN Tx)) and were subcutaneously infected with 0.1 mL of GAS suspension (~1 − 5 × 10^8^ CFU). No Inf control mice received 0.1 mL of PBS^−/−^. GAS-infected mice assigned to clindamycin (CLN, Gold Biotechnology, Olivette, MO, USA) treatment received the first dose of CLN (10 mg/kg/0.1 mL) administered intraperitoneally at 4–6 h after GAS infection. The regimen was then continued every day for an additional five days. Mice were monitored for survival, body weight, and lesions for sixteen days post-infection.

### 2.3. GAS Burden in the Blood, Brain, Skin, and the Subcutaneous Adipose Tissue

0.5–0.7 mL of terminal blood was drawn through cardiac puncture and collected into heparinized tubes (30U heparin, Akron Biotech, Boca Raton, FL, USA). The head was decapitated after blood collection, and the brain was excised rapidly. The left hemisphere was immersed in 4% paraformaldehyde, pH 7.4 (Thermo Fisher Scientific, Waltham, MA, USA) for fixation. The olfactory bulb and the cerebellum were removed from the right hemisphere. The hippocampi (HC) were isolated aseptically from the rest of the cerebral cortex (CC) and were collected into 1 mL sterile ice-cold PBS^−/−^. Necrotic skin and subcutaneous adipose tissue (SubQ AT) were recovered aseptically in 1 mL of sterile PBS^−/−^. Tissues were then weighed and homogenized (Omni International, Marietta, GA, USA), followed by plating of tenfold dilutions on blood agar plates for enumeration of bacterial load (calculated as colony-forming units (CFUs) per mL blood or milligram of tissue). The remaining homogenates were centrifuged for 15 min at 12,000× *g* at 4 °C, and supernatants were stored at −80 °C and used for cytokine profiling and western blot analysis.

### 2.4. Assessment of Cytokines in the Skin and the Brain Homogenates

Supernatants from the skin and cerebral homogenates were assayed using a fluorescence bead-based LEGENDplex Mouse Cytokine Panel (BioLegend, San Diego, CA, USA) as described [9]. This kit simultaneously quantifies 13 mouse cytokines, including IL-2, IL-4, IL-5, IL-6, IL-9, IL-10, IL-13, IL-17A, IL-17F, IL-21, IL-22, IFN-γ, and TNF-α. Assays were carried out according to the manufacturer’s instructions. Cytokine levels were detected by flow cytometry using BD-FACSymphony A3 (BD Biosciences, San Jose, CA, USA). Data were analyzed with the LEGENDplex™ software provided in the kit (version 8, BioLegend, San Diego, CA, USA).

### 2.5. Western Blot Detection of the Glial Fibrillary Acidic Protein (GFAP) and Ionized Calcium-Binding Adaptor-1 (Iba-1) Protein

Lysis buffer (2% *w*/*v* SDS, 6.25 mM Tris-HCl-7.5, 5mM EDTA) supplemented with protease (Thermo Fisher Scientific, Waltham, MA, USA) and phosphatase inhibitor cocktail (MilliporeSigma, Burlington, MA, USA) [35] was added to the HC and CC homogenates, and centrifuged at 14,000× *g* for 30 min at 4 °C. The supernatants were aliquoted and stored at −80 °C. Total protein concentration in the HC and CC supernatants was measured using the Pierce BCA Protein assay kit (Thermo Fisher Scientific). 50–100 µg of proteins were solubilized in a sample solubilizing buffer, heated for 10 min at 70 °C, and were resolved in Bis-Tris 4–20% SurePAGE gel (Genscript, Piscataway, NJ, USA). The proteins were electrophoretically transferred to the nitrocellulose membrane (Bio-Rad, Hercules, CA, USA). Membranes were blocked for one hour with Intercept protein-free blocking buffer (LI-COR, Lincoln, NE, USA) to avoid non-specific binding and were incubated overnight at 4 °C with primary antibodies against GFAP-1:1000, Cell Signalling Technology, Danvers, MA, USA; Iba-1-1:500, FujiFilm Wako, Richmond, VA, USA; and GAPDH-1:1000, Thermo Fisher Scientific, Waltham, MA, USA, prepared in Intercept blocking buffer (LI-COR) containing 0.2% Tween-20. The membranes were washed in Tris-buffered saline (TBS) containing 0.05% Tween-20 (TBST) and incubated in the dark for 2 h at room temperature with IRDye 680 goat anti-mouse and IRDye 800CW goat anti-rabbit (prepared 1:15,000 in Intercept blocking buffer containing 0.2% Tween-20). Blotted proteins were detected using the Odyssey CLx Imaging System (LI-COR). Western blots were analyzed using Fiji (a distribution of NIH ImageJ), and the average of the band densities was normalized to the loading control GAPDH.

### 2.6. GAS and Iba-1 Immunoreactivity

Paraformaldehyde fixed-left hemisphere of the brain from No Inf, No Tx, and CLN Tx mice were cryopreserved in 30% sucrose for 72 h and subsequently embedded in a Tissue-Tek^®^ O.C.T^TM^ compound (Thermo Fisher Scientific, Waltham, MA, USA). The brains were sectioned serially between bregma 2.80 mm and −4.24 mm using a cryostat (CM3050S, Leica Biosystems, Deer Park, IL, USA), and every sixth section of 40 μm thickness was collected into six-well plates containing PBS^−/−^. The brain sections were rinsed in PBS^−/−^ to remove O.C.T and were stored at 4 °C in a cryoprotectant solution of 30% ethylene glycol, 30% sucrose, and 0.02% sodium azide in PBS^−/−^. Serial brain sections were subjected to antigen retrieval (10 mM Tris, 1 mM EDTA, pH 9, at 95 °C for 10 min), rinsed in PBS^−/−^, and blocked with 5% goat serum containing 0.3% TritonX-100 in PBS^−/−^ for 1 h at room temperature. The sections were incubated overnight at 4 °C with the primary antibody anti-Group A Streptococcus (1:100, Thermo Fisher Scientific, Waltham, MA, USA) or anti-Iba-1 (1:1000) [36]. The following day, the sections were rinsed and incubated with secondary goat anti-rabbit IgG-AF594 antibody (1:1000, Thermo Fisher Scientific, Waltham, MA, USA) for 2 h at room temperature in the dark. Sections were rinsed and counterstained with DAPI (MilliporeSigma, Burlington, MA, USA), mounted onto gelatin-coated glass slides, dried overnight, and coverslipped with Fluoromount (MilliporeSigma, Burlington, MA, USA).

### 2.7. Image Acquisition

Immunodetection of GAS bacteria in the brain was performed using an Olympus Fluoview 3000 confocal laser scanning microscope coupled with an Olympus IX83 microscope (Olympus, Waltham, MA, USA). Z-stack images with 2.5× zoom were obtained using a 60× oil-immersion objective lens (PlanApo N 60×/1.40 oil SC ∞/0.17/FN22). All the images were captured with a constant 4.24% laser intensity, 502 HV sensitivity, 1% gain, and 0 offset for DAPI at 405 nm and with 4.42% laser intensity, 584 HV sensitivity, 1% gain, and 0 offset for anti-GAS at 594 nm, with a setup step size of 1.0 μm. Consecutive cross-section images (XYZ) were acquired from top to bottom; after the acquisition, projections of z-stack images were displayed using the Z mode.

For immunodetection of Iba-1 cells, the images were captured at 20× magnification (×20/0.4 HC PL Fluotar, CORR PH1 lens) on a DMi8 microscope (Leica THUNDER Imager, Leica Microsystems, Deer Park, IL, USA) connected to a high-resolution CMOS camera (up to ~1000 fps) using the computational clearing feature in the Leica Application Suite X software 3.7.2.22383 (Leica Microsystems, Wetzlar, Germany). Iba-1 immunoreactivity was quantified using images from two sections per mouse and at least three mice per condition using Fiji (a distribution of NIH ImageJ). Subregions of the images were selected using the marque ROI tool, set to 8-bit, preprocessed by adjusting brightness and contrast to facilitate segmentation, and then segmented and counted using a batch-processing macro that incorporated a machine learning segmentation plugin (Trainable Weka Segmentation) [37] and the Analyze Particles feature in Fiji [38,39,40]. Before the analysis, the machine learning algorithm was trained by manually defining labeled and unlabeled regions in selected representative images and assigning them to separate classes. The resulting classifier file was then used in the analysis.

### 2.8. Statistical Analysis

Values are presented as mean ± SEM and were analyzed using the two-tailed Student’s *t*-test and one-way or two-way analysis of variance (ANOVA) followed by uncorrected Fisher’s LSD test under a statistical threshold of *p* < 0.05 using Prism^®^ 10.0.2 (GraphPad Software Inc. San Diego, CA, USA).

## 3. Results

### 3.1. Clindamycin Treatment Significantly Reduced Weight Loss and Lesion Areas following Subcutaneous GAS Infection

**Figure 1:**
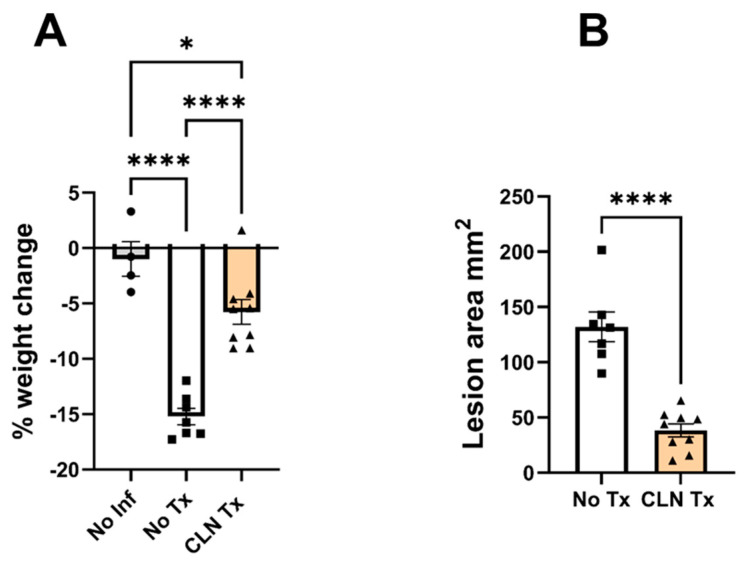
CLN treatment significantly reduced weight loss and lesion areas. DQ8 mice were infected subcutaneously with 1 − 5 × 10^8^ CFU of GAS 5448 and were either untreated or treated with CLN (10 mg/kg) administered intraperitoneally every day for five days. Control mice were uninfected (No Inf). Body weight and skin lesion area were measured, and data are presented as mean values + SEM (*n* = 4–9). Each circle, square, or triangle represents an individual mouse. Statistical differences were computed by one-way ANOVA followed by uncorrected Fisher’s LSD test ((**A**), * *p* = 0.0338, and **** *p* < 0.0001) or unpaired *t*-test ((**B**), **** *p* < 0.0001).

Our laboratory has developed mouse models of subcutaneous GAS infections in outbred [33], ARI BXD [34], and clinically relevant humanized mice expressing HLA-II alleles associated with varying susceptibility to GAS NSTI/STSS [9]. Here, we studied the effect of CLN treatment on survival, changes in body weight, skin lesions, GAS burden in the skin, SubQ AT, and HC and CC regions of the brain following subcutaneous GAS infection in DQ8 mice. There was no mortality associated with subcutaneous GAS infection in the DQ8 mice. Body weight loss has been established as a significant comorbidity associated with poor recovery from subcutaneous GAS infections [33,34]. As shown in Figure 1A, compared to uninfected mice (No Inf), weight loss was significant following subcutaneous GAS infection (**** *p* < 0.0001) (No Tx). Consistent with the significant reduction in weight loss (No Tx vs. CLN Tx **** *p* < 0.0001; No Inf vs. CLN Tx: * *p* = 0.0338), CLN treatment also resulted in significant amelioration of lesions in DQ8 mice (No Tx vs. CLN Tx **** *p* < 0.0001) (Figure 1B).

### 3.2. Heterogeneity in GAS Burden in the Skin, Subcutaneous Adipose Tissue, and the Brain

**Figure 2:**
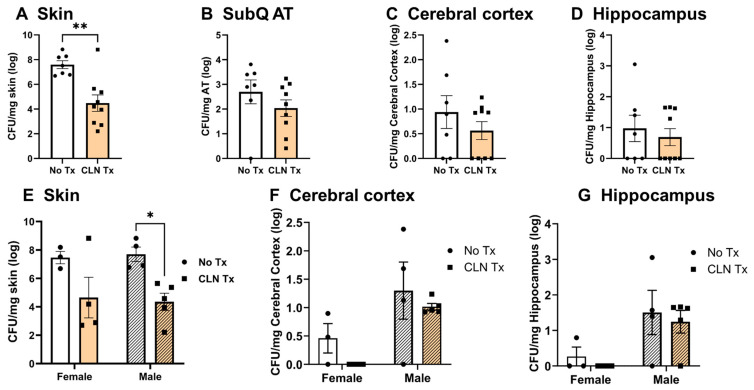
Heterogeneity in GAS burden in the skin, subcutaneous adipose tissue, and the brain. DQ8 mice were infected subcutaneously with 1 − 5 × 10^8^ CFU of GAS 5448 and were either untreated or treated with CLN (10 mg/kg) administered intraperitoneally every day for five days. Sixteen days post-infection, necrotic skin lesions, SubQ AT, the right cerebral cortex, and hippocampus were aseptically excised and collected into 1 mL of sterile PBS^−/−^, weighed, and homogenized to enumerate the GAS burden ((**A**–**D**) respectively). GAS burden in female and male mice was compared separately in the skin (**E**), cerebral cortex (**F**), and hippocampus (**G**). Data are presented as mean values + SEM (*n* = 4–9). Each circle or square represents an individual mouse. Statistical differences were computed by unpaired *t*-test ((**A**), ** *p* = 0.0018) or two-way ANOVA ((**E**), * *p* = 0.0140).

We next investigated the GAS burden in the blood, skin, SubQ AT, and brain. At sixteen days post-infection, GAS was not detectable in the blood of DQ8 mice. As shown in Figure 2A, No Tx mice showed a significantly higher GAS burden in the skin compared with the CLN Tx group (** *p* = 0.0018). GAS disseminated into SubQ AT, and CLN treatment reduced SubQ AT GAS burden; however, the difference was insignificant (Figure 2B). Most strikingly, GAS translocated into the CC (Figure 2C) and HC (Figure 2D) regions of the brain. The GAS burden persisted in the brain sub-regions after CLN treatment (Figure 2C,D). To determine whether there was a sex difference in GAS burden, CFUs from female and male mice were compared separately. As shown in Figure 2E–G, CLN effectively reduced the GAS burden in the skin (Figure 2E, * *p* = 0.0140) of male mice but not in the CC or HC of female or male DQ8 mice.

### 3.3. GAS Bacteria Localize to the Brain

**Figure 3:**
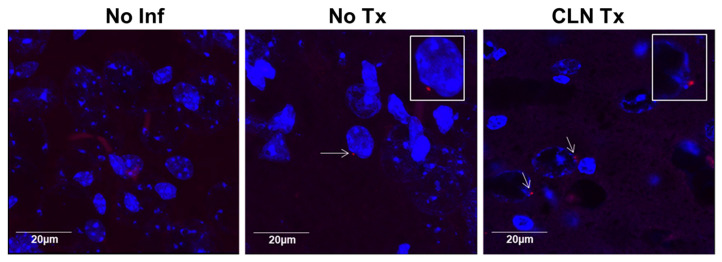
GAS bacteria localize to the brain.

The dissemination of GAS into the brain following peripheral skin infection presents a novel situation that was unknown previously. Our data demonstrate remarkable sex-dependent differences in GAS burden in the brain following subcutaneous GAS infection. Z-stacked images of coronal brain sections stained with anti-GAS antibodies demonstrate the presence of GAS in the vicinity of the neuronal nucleus in the neocortex of both GAS-infected untreated and CLN-treated male DQ8 mice (Figure 3).

Representative images showing immunoreactivity of the GAS antibody in the cerebral cortex of No Inf, No Tx, or CLN Tx DQ8 male mice. The 40μm thick sections of the brain (left hemisphere) were stained with rabbit polyclonal anti-GAS antibody (1:100) followed by Alexa Fluor 594-conjugated goat anti-rabbit secondary antibody (1:1000) (Red). Nuclei were counterstained with DAPI. Images were captured using an Olympus FV3000 confocal laser scanning microscope coupled with an Olympus IX83 microscope at 60x magnification with 2.5× Zoom (scale = 20 µm). The arrows in No Tx and CLN Tx panels point to positive GAS immunoreactivity localized near the nucleus. Magnified insets in panels No Tx and CLN Tx show GAS bacteria in close proximity to cell nuclei.

### 3.4. Clindamycin Treatment Significantly Altered the Cytokine Profile in the Skin and Cerebral Cortex

**Figure 4:**
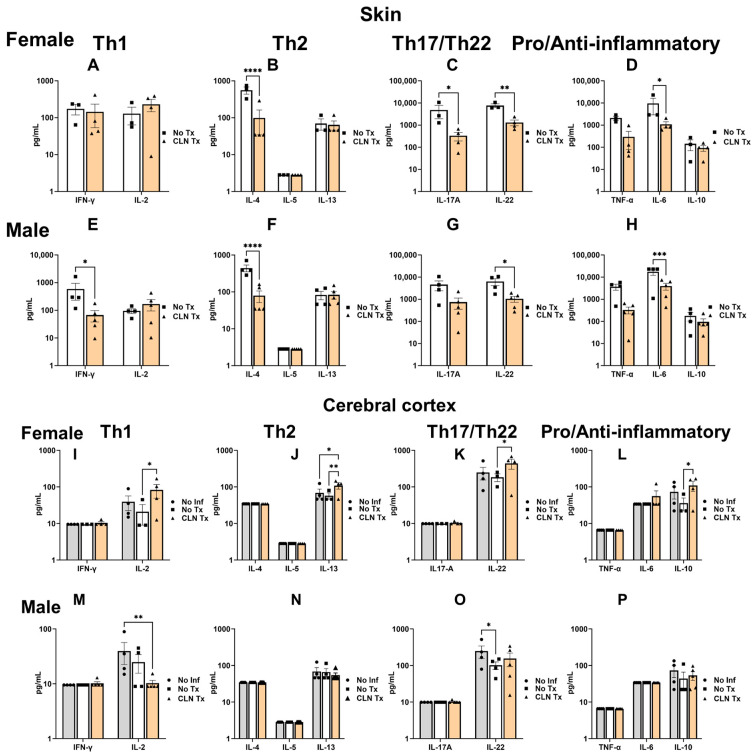
Clindamycin treatment significantly altered the cytokine profile in the skin and cerebral cortex. DQ8 mice were infected subcutaneously with 1 − 5 × 10^8^ CFU of GAS 5448 and were either untreated or treated with CLN (10 mg/kg) administered intraperitoneally every day for five days. Sixteen days post-infection, homogenates from the skin and cerebral cortex were used to analyze a panel of Th1 (IFN-γ, IL-2), Th2 (IL-4, IL-5, IL-13), Th17/Th22 (IL-17A, IL-22), pro (TNF-α and IL-6), and anti-inflammatory (IL-10) cytokines using the BioLegend LegendPlex Assay. Panels (**A**–**D**): Th1, Th2, Th17/Th22, and Pro/Anti-inflammatory cytokine families in female skin; Panels (**E–H**): Th1, Th2, Th17/Th22, and Pro/Anti-inflammatory cytokine families in male skin; Panels (**I**–**L**): Th1, Th2, Th17/Th22, and Pro/Anti-inflammatory cytokine families in the female cerebral cortex; Panels (**M**–**P**): Th1, Th2, Th17/Th22, and Pro/Anti-inflammatory cytokine families in the male cerebral cortex. Data are presented as mean values ± SEM of at least three biological replicates. Each circle, square, or triangle represents an individual mouse. Statistical differences were computed by two-way ANOVA followed by uncorrected Fisher’s LSD test. **** *p* < 0.0001, IL-4; * *p* = 0.0442, IL-17 (**A**); ** *p* = 0.0092, IL-22; * *p* = 0.0192, IL-6; (**B**–**D**); * *p* = 0.0405, IFN-γ; **** *p* < 0.0001, IL-4; * *p* = 0.0172, IL-22; *** *p* = 0.0002, IL-6; (**E**–**H**); No Tx Vs. CLN Tx: * *p =* 0.0280, IL-2; ** *p =* 0.0040, IL-13; * *p =* 0.0340, IL-22; * *p =* 0.0108, IL-10; No Inf Vs CLN Tx: * *p =* 0.0130, IL-13, (**I**–**L**); ** *p* = 0.0092, IL-2, (**M**); and * *p* = 0.0462, IL-22, (**O**)).

We next wanted to investigate whether the significant reduction in lesion size and reduced GAS burden in the skin but not the brain following CLN treatment would correlate with changes in the cytokine profile. We assessed the levels of multiple prototypical Th1 (IFN-γ, IL-2), Th2 (IL-4, IL-5, IL-13), Th17/Th22 (IL-17A, IL-22), pro (TNF-α and IL-6), and anti-inflammatory (IL-10) cytokines as dominant readouts of immune modulation. The levels of IL-4, IL-17A, IL-22, and IL-6 were significantly reduced in the skin homogenates of CLN Tx female mice compared to the No Tx group (No Tx vs. CLN Tx: **** *p* < 0.0001, IL-4; * *p* = 0.0442, IL-17A; ** *p* = 0.0092, IL-22; * *p* = 0.0192, IL-6; Figure 4B–D). In males, the levels of IFN-γ, IL-4, IL-22, and IL-6 were significantly reduced in the CLN Tx group compared to the No Tx group (No Tx vs. CLN Tx: * *p* = 0.0405, IFN-γ; **** *p* < 0.0001, IL-4; * *p* = 0.0172, IL-22; *** *p* = 0.0002, IL-6; Figure 4E–H). Next, we investigated whether GAS dissemination into the brain or peripheral inflammatory cytokines communicated to the brain and altered the brain cytokine profile. As shown in Figure 4I–L, there was a significant increase in the levels of IL-2, IL-22, and IL-10 in the cerebral cortex homogenates of CLN Tx female mice compared to the No Tx group, while IL-13 levels were significantly upregulated compared to both No Inf and No Tx groups (No Tx vs. CLN Tx: * *p =* 0.0280, IL-2; ** *p =* 0.0040, IL-13; * *p =* 0.0340, IL-22; * *p =* 0.0108, IL-10; No Inf vs. CLN Tx: * *p =* 0.0130, IL-13). In the males, however, cortical IL-2 levels were significantly reduced in the CLN Tx group compared to the No Inf group (Figure 4M). Compared to the No Inf group, IL-22 levels were significantly reduced in the No Tx group, which did not improve with CLN treatment (Figure 4O) (No Inf vs. CLN Tx: ** *p* = 0.0092, IL-2; and No Inf vs. No Tx: * *p* = 0.0462, IL-22).

### 3.5. Subcutaneous GAS Infection Increased Cortical and Hippocampal Iba-1 Protein Levels

**Figure 5:**
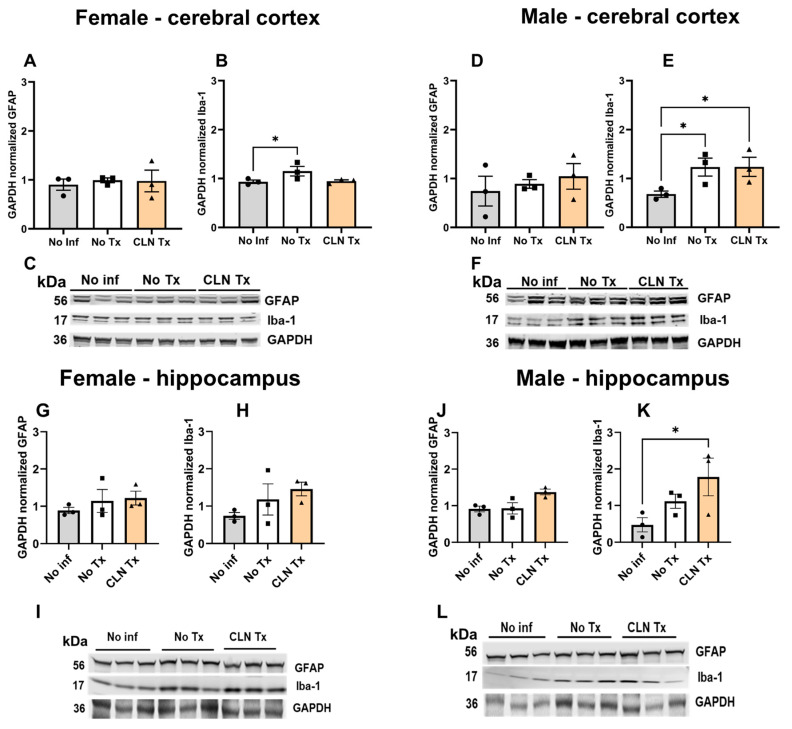
Subcutaneous GAS infection increased cortical and hippocampal Iba-1 protein levels. DQ8 mice were infected subcutaneously with 1 − 5 × 10^8^ CFU of GAS 5448 and were either untreated or treated with CLN (10 mg/kg) administered intraperitoneally every day for five days. Sixteen days post-infection, cerebral cortex and hippocampal homogenates were lysed and resolved by SDS-PAGE for western blot analysis using antibodies against GFAP, Iba-1, and GAPDH (loading control). GAPDH normalized values are expressed as mean values ± SEM. Each circle, square, or triangle represents an individual mouse. One-way ANOVA followed by uncorrected Fisher’s LSD test was used to determine statistical significance. Panels: (**A**): GAPDH normalized GFAP (female, cerebral cortex); (**B**): GAPDH normalized Iba-1 (female, cerebral cortex, * *p* = 0.0477 Female No Inf vs. No Tx); (**C**): Representative western blot GFAP, Iba-1, and GAPDH (female, cerebral cortex); (**D**): GAPDH normalized GFAP (male, cerebral cortex); (**E**): GAPDH normalized Iba-1 (male, cerebral cortex, * *p* = 0.0487 Male No Inf vs. No Tx, * *p* = 0.0471, No Inf vs. CLN Tx); (**F**): Representative western blot GFAP, Iba-1, and GAPDH (male, cerebral cortex); (**G**): GAPDH normalized GFAP (female, hippocampus); (**H**): GAPDH normalized Iba-1 (female, hippocampus); (**I**): Representative western blot GFAP, Iba-1, and GAPDH (female, hippocampus); (**J**): GAPDH normalized GFAP (male, hippocampus); (**K**): GAPDH normalized Iba-1 (male, hippocampus, * *p* = 0.0338 No Inf vs. CLN Tx); (**L**): Representative western blot GFAP, Iba-1, and GAPDH (male, hippocampus).

GFAP is the characteristic intermediate filament protein of astrocytes and is an important neuroinflammation marker [41,42]. Iba-1 is widely used as a pan-microglial marker to identify and quantify microglia in the brain. Increased Iba-1 levels have been correlated with inflammatory states, as their levels are dramatically upregulated in response to various neuroinflammatory stimuli [43,44]. We assessed the protein levels of GFAP and Iba-1 to quantify glial changes in the cortical and hippocampal lysates. While GFAP protein levels were not altered, there was a significant increase in the cortical Iba-1 levels in the female and male No Tx group compared to No Inf controls (Figure 5B, * *p* = 0.0477 Female No Inf vs. No Tx); Figure 5E, * *p* = 0.0487 Male No Inf vs. No Tx), suggestive of a microglial response to subcutaneous GAS infection. Correlating with the persistence of GAS despite CLN treatment in the male mice, Iba-1 levels also remained significantly elevated in CLN Tx male mice compared to No Inf controls (Figure 5E * *p* = 0.0471, No Inf vs. CLN treatment) in the cortex, and in the hippocampus (Figure 5K, * *p* = 0.0338 No Inf vs. CLN Tx).

### 3.6. Subcutaneous GAS Infection Increased Iba-1 Immunoreactivity in Male Mice

**Figure 6:**
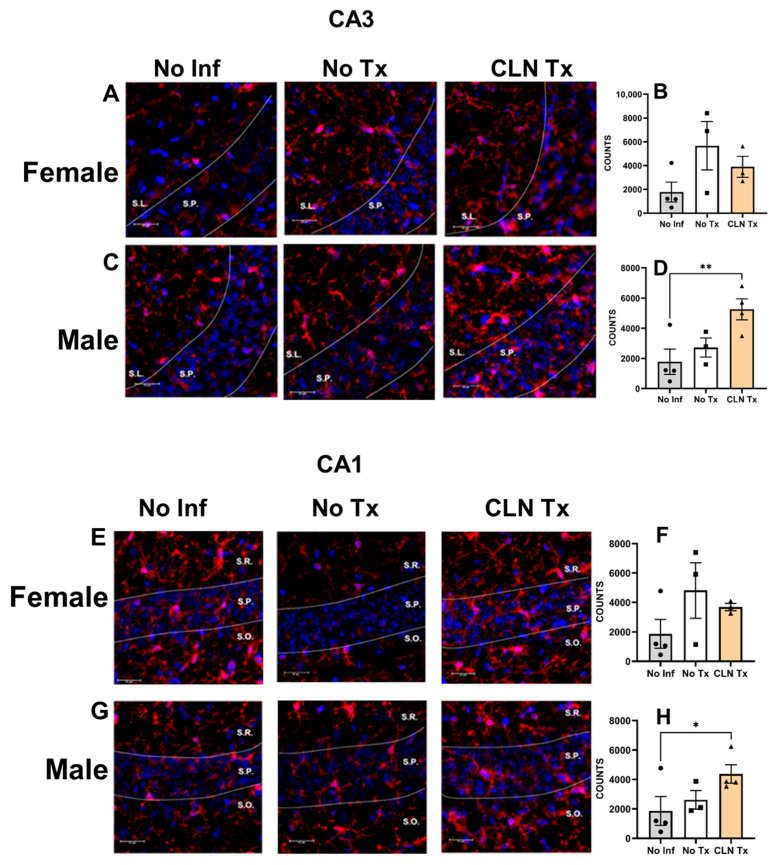

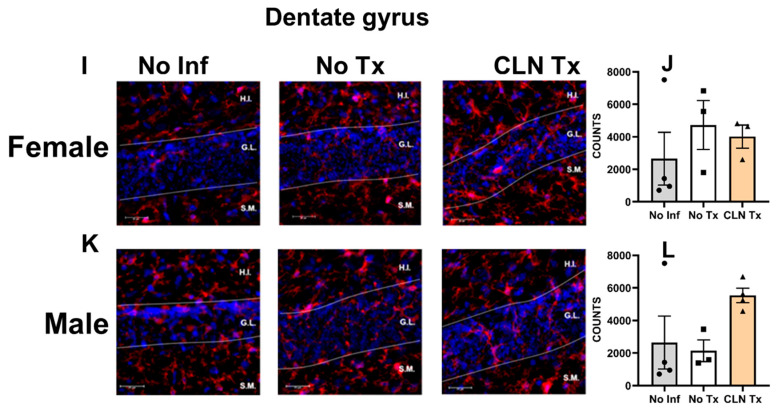
Subcutaneous GAS infection increased Iba-1 immunoreactivity in the male mice. DQ8 mice were infected subcutaneously with 1 − 5 × 10^8^ CFU of GAS 5448 and were either untreated or treated with CLN (10 mg/kg) administered intraperitoneally every day for five days. Sixteen days post-infection, the left hemisphere of the brain was fixed and serial sectioned (40 µm thickness). Every sixth section was stained with rabbit polyclonal anti-Iba-1 (1:1000), followed by Alexa Fluor 594-conjugated goat anti-rabbit secondary antibody (1:1000) (Red). The nuclei were stained with DAPI, and images were captured using the Leica THUNDER Imager at 20× magnification. Panels (**A**) and (**C**) (CA3), (**E**) and (**G**) (CA1), and (**I**) and (**K**) (Dentate gyrus) show representative images (scale bar = 25 µm) of Iba-1 immunoreactivity in the brain tissue from No Inf, No Tx, or CLN Tx mice. Bars represent mean values ± SEM of segmented counts of Iba-1 positive cells (*n* = 3–4 per group). Each circle, square, or triangle represents an individual mouse. One-way ANOVA followed by uncorrected Fisher’s LSD test was used to determine statistical significance. Panels: (**A**): CA3 Female (No Inf, No Tx, CLN Tx; S.P. stratum pyramidale, S.L. stratum lucidum); (**B**): Mean values ± SEM of segmented counts of Iba-1 positive cells in CA3 female; (**C**): CA3 male (No Inf, No Tx, CLN Tx; S.P. stratum pyramidale, S.L. stratum lucidum); (**D**): Mean values ± SEM of segmented counts of Iba-1 positive cells in CA3 male, ** *p* = 0.0091); (**E**): CA1 female (No Inf, No Tx, CLN Tx; S.O. stratum oriens, S.P stratum pyramidale, S.R. stratum radiatum); (**F**): Mean values ± SEM of segmented counts of Iba-1 positive cells in CA1 female; (**G**): CA1 male (No Inf, No Tx, CLN Tx; S.O. stratum oriens, S.P stratum pyramidale, S.R. stratum radiatum); (**H**): Mean values ± SEM of segmented counts of Iba-1 positive cells in CA1 male, * *p* = 0.0491); (**I**): Dentate gyrus female (No Inf, No Tx, CLN Tx; S.M. stratum moleculare, G.L. granule cell layer, H.I. hilus of dentate gyrus); (**J**): Mean values ± SEM of segmented counts of Iba-1 positive cells in Dentate gyrus female; (**K**): Dentate gyrus male (No Inf, No Tx, CLN Tx; S.M. stratum moleculare, G.L. granule cell layer, H.I. hilus of dentate gyrus); (**L**): Mean values ± SEM of segmented counts of Iba-1 positive cells in Dentate gyrus male.

Since peripheral GAS infections selectively induced Iba-1 protein levels in the hippocampal lysates, we assessed Iba-1 immunoreactivity in the HC. Our data show that subcutaneous GAS infections significantly increased Iba-1 reactivity in the CA3 (Figure 6A–D) and CA1 (Figure 6E–H) subregions of CLN Tx male mice compared to No Inf controls (No Inf vs. CLN Tx, ** *p* = 0.0091, CA3 (Figure 6D)); * *p* = 0.0491, CA1 (Figure 6H). However, the female mice had no such differences (Figure 6A,B,E,F). There were no differences in the Iba-1 immunoreactivity among the groups in the females or males in the dentate gyrus (Figure 6I–L).

## 4. Discussion

There has been a growing interest in understanding the role of bacterial or bacterial product-induced peripheral immune responses as a causative agent of neuroinflammation, neurodegeneration, and related cognitive dysfunction [45,46,47,48]. Systemic exposure to gram-negative bacterial endotoxin, LPS, has been widely used to demonstrate neuroinflammation and associated sickness behavior [49]. Liu et al. reported that subcutaneous infections with the GAS M28 serotype in female NF-κΒ reporter mice cause peripheral TNF-mediated neuroinflammation that can be attenuated by administering a dominant-negative selective inhibitor of TNF [50].

The ability of GAS to cross the blood–brain barrier or the meninges and reach the brain following a skin infection has not been demonstrated. Our results show that female mice harbored less brain GAS burden than males despite comparable GAS burden and CLN-mediated resolution in the skin. The lower initial brain GAS burden was, perhaps, more readily cleared by CLN in the female mice, in contrast to persistence observed in the male mice. The sex-dependent and compartmentalized antibiotic responses are intriguing and remain to be investigated. These data suggest that the brain constitutes a viable niche for GAS despite CLN intervention and might serve as an endogenous source for reactivation or relapse. While we have not been able to detect free bacteria in the brain, we could identify likely neuronal cells that have internalized GAS bacteria. Our findings raise the possibility that CLN, a bacteriostatic macrolide and the standard line of therapy for invasive GAS infections [51,52], may have limited ability to eliminate intracellular GAS reservoirs sequestered within brain cells. It is established that host mimics of GAS proteins result in anti-dopaminergic autoantibodies through molecular mimicry affecting dopamine signaling pathways [53]. However, whether GAS produces adhesins or other host mimics that can target neuronal receptors is unknown. We cannot exclude other cell types in the brain as targets, and they need to be identified. Further, the mechanism of GAS invasion, including the indirect “trojan horse mechanism,” through riding in GAS-infected peripherally derived macrophages and spreading from infected macrophages to neurons remains to be elucidated.

The therapeutic efficiency of CLN was evident based on the significant suppression of IFN-γ, IL-4, IL-17A, IL-22, and IL-6 levels in the skin homogenates from CLN Tx mice, which correlated with reduced lesion size. Specifically, our data indicate that the IL-6/Th17/Th22 axis is crucial to the pathogenesis of subcutaneous GAS infections. It is well-established that IL-6 plays a critical role at multiple levels in polarizing the differentiation of naïve CD4^+^ T cells into IL-17/IL-22-secreting effector Th17/Th22 subsets through direct and indirect mechanisms [54]. We predicted that the exaggerated local inflammatory response, a Th17/Th22 axis driven by IL-6 levels, can transmit signals to the brain that might profoundly affect the cytokine profile and neuroimmune responses. CLN is considered a first-line treatment choice in the clinical management of invasive GAS infections [55,56]; however, the sex-dependent effects of CLN have not been widely examined in invasive GAS infections. Our data demonstrating the coordinated upregulation in the cortical levels of IL-2, IL-13, IL-22, and IL-10 in CLN Tx female mice that lacked GAS burden suggests that CLN triggers a concerted shift toward cytokines recognized to exert immunosuppressive effects [57,58]. Interestingly, in the males, where cortical and hippocampal GAS burden was observed, IL-2 levels were significantly reduced in the CLN Tx group compared to the No Inf group. IL-2, traditionally recognized for its ability to support T cell activation and proliferation, conversely tends to dampen inflammation by augmenting T regulatory cell numbers [59]. Whether IL-2 signaling appears poised to exert divergent outcomes under conditions of exposure to GAS in the brain remains to be examined.

Our data show that cortical IL-22 levels were significantly reduced in the male No Tx group compared to the No Inf group. Type 3 innate lymphoid cells (ILC3) are the primary source of IL-22, a member of the IL-10 family, and a key cytokine involved in maintaining intestinal barrier integrity [60]. Interestingly, ILC3 cells can be stimulated by enteric glial cells to produce more IL-22 and regulate brain and cognitive function [61,62]. Further work is needed to establish whether streptococcal skin infections induce intestinal damage or inflammation like colitis and whether increased IL-22 in the brain following CLN treatment is an unintended consequence of CLN therapy, causing gut dysbiosis and alterations through the bidirectional gut–brain axis. In addition to ILC3 cells, the Th22 subset of CD4^+^ cells is a primary source of IL-22 during infections [63,64]. Liang et al. reported that TGF-β initiates the cascade of pro-inflammatory cytokines IL-17A, IL-22, and IL-6, and together, IL-17A and IL-22 are associated with skin innate immune responses [65]. Our study suggests the possibility that subcutaneous GAS infection-induced IL-22 assumes a pathogenic role mirroring IL-17A levels in the skin and a protective role in the brain mirroring IL-10 levels. Our future studies will assess the dichotomous role of IL-22 in the skin–brain axis during GAS infections.

Insults in the brain due to trauma, infections, and peripheral inflammatory mediators prime the microglia for activation that correlates with the release of pro-inflammatory mediators, chemokines, and free radicals and can lead to the distinct polarization of a pro- or anti-inflammatory phenotype depending on the type of insult [66]. In the case of pro-inflammatory response, mediators released by activated microglia can, in turn, confer an activated state in responding astrocytes [67]. Together, these reactive microglia and astrocytes can potentiate neuron and synapse loss, the progression of neurodegeneration, and chronic neuroinflammation [68]. Direct evidence for the role of activated microglia and astrocytes in inducing inflammatory mediators leading to neuroinflammation and cognitive impairment has been established due to HIV-1 infections [69,70,71,72]. Our western blot data showed that subcutaneous GAS infections significantly increased the protein levels of the microglia marker, Iba-1, in the CC of female and male mice and HC of male mice. The hippocampus is highly susceptible to changes in a redox state [73], age [74], or pathogens [75] and demonstrates a unique selective neuronal vulnerability [76]. In the present study, we observed concerning findings regarding the therapeutic failure of CLN to eliminate brain GAS burden in the HC that coincided with CA3 and CA1 subregion selective changes in Iba-1 immunoreactivity in the male mice. We recognize that an important limitation of the present study was the use of Iba-1 as the primary marker to characterize microglia without the use of additional markers associated with an activated phenotype and functional status. Nonetheless, our current findings provide an initial framework for more detailed follow-up investigations. They are designed to include additional markers for robust microglia profiling to elucidate their protective and pathogenic roles during GAS infections.

## 5. Conclusions

In conclusion, our data show that despite comparable resolution of skin GAS burden and skin cytokine changes following CLN therapy in GAS-infected female and male DQ8 mice, an altered brain cytokine profile emerged in CLN Tx mice that was sex-dependent. GAS dissemination into the brain and microglial responses were also sex-dependent and subregion-specific. These results open new avenues for detailed investigations, including (a) antibiotic combinations, (b) host-directed therapies as antibiotic adjuncts, (c) mechanisms underlying the sexual dimorphism in GAS invasion and antibiotic responses within the CNS, (d) receptors targeted for intracellular GAS persistence within brain cells, and (e) long-term functional and behavioral consequences of infections caused by GAS, one of the most important human pathogens with the broadest spectrum of clinical manifestations known in medical history.

## Data Availability

All data are included in the published article.

## References

[1] Cunningham M.W. (2000). Pathogenesis of Group A Streptococcal Infections. Clin. Microbiol. Rev..

[2] Aziz R.K., Kotb M. (2008). Rise and Persistence of Global M1T1 Clone of Streptococcus Pyogenes. Emerg. Infect. Dis..

[3] Henningham A., Barnett T.C., Maamary P.G., Walker M.J. (2012). Pathogenesis of Group A Streptococcal Infections. Discov. Med..

[4] Nitzsche R., Rosenheinrich M., Kreikemeyer B., Oehmcke-Hecht S. (2015). Streptococcus Pyogenes Triggers Activation of the Human Contact System by Streptokinase. Infect. Immun..

[5] Kotb M., Norrby-Teglund A., McGeer A., El-Sherbini H., Dorak M.T., Khurshid A., Green K., Peeples J., Wade J., Thomson G. (2002). An Immunogenetic and Molecular Basis for Differences in Outcomes of Invasive Group A Streptococcal Infections. Nat. Med..

[6] Nooh M.M., Nookala S., Kansal R., Kotb M. (2011). Individual Genetic Variations Directly Effect Polarization of Cytokine Responses to Superantigens Associated with Streptococcal Sepsis: Implications for Customized Patient Care. J. Immunol..

[7] Kotb M., Norrby-Teglund A., McGeer A., Green K., Low D.E. (2003). Association of Human Leukocyte Antigen with Outcomes of Infectious Diseases: The Streptococcal Experience. Scand. J. Infect. Dis..

[8] Bessen D.E. (2016). Tissue Tropisms in Group A Streptococcus: What Virulence Factors Distinguish Pharyngitis from Impetigo Strains?. Curr. Opin. Infect. Dis..

[9] Nookala S., Mukundan S., Fife A., Alagarsamy J., Kotb M. (2018). Heterogeneity in FoxP3- and GARP/LAP-Expressing T Regulatory Cells in an HLA Class II Transgenic Murine Model of Necrotizing Soft Tissue Infections by Group A Streptococcus. Infect. Immun..

[10] Cutforth T., Demille M.M.C., Agalliu I., Agalliu D. (2016). CNS Autoimmune Disease after Streptococcus Pyogenes Infections: Animal Models, Cellular Mechanisms and Genetic Factors. Future Neurol..

[11] Martino D., Defazio G., Giovannoni G. (2009). The PANDAS Subgroup of Tic Disorders and Childhood-Onset Obsessive-Compulsive Disorder. J. Psychosom. Res..

[12] Martino D., Chiarotti F., Buttiglione M., Cardona F., Creti R., Nardocci N., Orefici G., Veneselli E., Rizzo R. (2011). The Relationship between Group A Streptococcal Infections and Tourette Syndrome: A Study on a Large Service-Based Cohort. Dev. Med. Child Neurol..

[13] Murphy T.K., Petitto J.M., Voeller K.K., Goodman W.K. (2001). Obsessive Compulsive Disorder: Is There an Association with Childhood Streptococcal Infections and Altered Immune Function?. Semin. Clin. Neuropsychiatry.

[14] Vezzani A., Fujinami R.S., White H.S., Preux P.M., Blümcke I., Sander J.W., Löscher W. (2016). Infections, Inflammation and Epilepsy. Acta Neuropathol..

[15] Nielsen H., Storgaard M., Helweg-Larsen J., Larsen L., Jepsen M.P.G., Hansen B.R., Wiese L., Bodilsen J. (2023). Group A Streptococcus Meningitis in Adults, Denmark. Emerg. Infect. Dis..

[16] Dehority W., Uchiyama S., Khosravi A., Nizet V. (2006). Brain Abscess Caused by Streptococcus Pyogenes in a Previously Healthy Child. J. Clin. Microbiol..

[17] Hayashi A., Takano T., Suzuki A., Narumiya S. (2011). Group A Streptococcal Brain Abscess: A Case Report and a Review of the Literature since 1988. Scand. J. Infect. Dis..

[18] Sheeler C., Rosa J.G., Ferro A., McAdams B., Borgenheimer E., Cvetanovic M. (2020). Glia in Neurodegeneration: The Housekeeper, the Defender and the Perpetrator. Int. J. Mol. Sci..

[19] Vainchtein I.D., Molofsky A.V. (2020). Astrocytes and Microglia: In Sickness and in Health. Trends Neurosci..

[20] Nooh M.M., El-Gengehi N., Kansal R., David C.S., Kotb M. (2007). HLA Transgenic Mice Provide Evidence for a Direct and Dominant Role of HLA Class II Variation in Modulating the Severity of Streptococcal Sepsis. J. Immunol..

[21] Ascough S., Ingram R.J., Chu K.K., Reynolds C.J., Musson J.A., Doganay M., Metan G., Ozkul Y., Baillie L., Sriskandan S. (2014). Anthrax Lethal Factor as an Immune Target in Humans and Transgenic Mice and the Impact of HLA Polymorphism on CD4+ T Cell Immunity. PLoS Pathog..

[22] Nabozny G.H., Baisch J.M., Cheng S., Cosgrove D., Griffiths M.M., Luthra H.S., David C.S. (1996). HLA-DQ8 Transgenic Mice Are Highly Susceptible to Collagen-Induced Arthritis: A Novel Model for Human Polyarthritis. J. Exp. Med..

[23] Taneja V., David C.S. (2010). Role of HLA Class II Genes in Susceptibility/Resistance to Inflammatory Arthritis: Studies with Humanized Mice. Immunol. Rev..

[24] Taneja V., Taneja N., Paisansinsup T., Behrens M., Griffiths M., Luthra H., David C.S. (2002). CD4 and CD8 T Cells in Susceptibility/Protection to Collagen-Induced Arthritis in HLA-DQ8-Transgenic Mice: Implications for Rheumatoid Arthritis. J. Immunol..

[25] Germundson D.L., Nookala S., Smith N.A., Warda Y., Nagamoto-Combs K. (2022). HLA-II Alleles Influence Physical and Behavioral Responses to a Whey Allergen in a Transgenic Mouse Model of Cow’s Milk Allergy. Front. Allergy.

[26] Neeno T., Krco C.J., Harders J., Baisch J., Cheng S., David C.S. (1996). HLA-DQ8 Transgenic Mice Lacking Endogenous Class II Molecules Respond to House Dust Allergens: Identification of Antigenic Epitopes. J. Immunol..

[27] Pavelko K.D., Drescher K.M., McGavern D.B., David C.S., Rodriguez M. (2000). HLA-DQ Polymorphism Influences Progression of Demyelination and Neurologic Deficits in a Viral Model of Multiple Sclerosis. Mol. Cell. Neurosci..

[28] Mangalam A.K., Luo N., Luckey D., Papke L., Hubbard A., Wussow A., Smart M., Giri S., Rodriguez M., David C. (2014). Absence of IFN-γ Increases Brain Pathology in Experimental Autoimmune Encephalomyelitis–Susceptible DRB1*0301.DQ8 HLA Transgenic Mice through Secretion of Proinflammatory Cytokine IL-17 and Induction of Pathogenic Monocytes/Microglia into the Central Nerv. J. Immunol..

[29] Mangalam A., Luckey D., Basal E., Jackson M., Smart M., Rodriguez M., David C. (2009). HLA-DQ8 (DQB1*0302)-Restricted Th17 Cells Exacerbate Experimental Autoimmune Encephalomyelitis in HLA-DR3-Transgenic Mice. J. Immunol..

[30] Ambigapathy G., Mukundan S., Nagamoto-Combs K., Combs C.K., Nookala S. (2023). HLA-II-Dependent Neuroimmune Changes in Group A Streptococcal Necrotizing Fasciitis. Pathogens.

[31] Knopick P., Terman D., Riha N., Alvine T., Larson R., Badiou C., Lina G., Ballantyne J., Bradley D. (2020). Endogenous HLA-DQ8αβ Programs Superantigens (SEG/SEI) to Silence Toxicity and Unleash a Tumoricidal Network with Long-Term Melanoma Survival. J. Immunother. Cancer.

[32] Chatellier S., Ihendyane N., Kansal R.G., Khambaty F., Basma H., Norrby-Teglund A., Low D.E., McGeer A., Kotb M. (2000). Genetic Relatedness and Superantigen Expression in Group A Streptococcus Serotype M1 Isolates from Patients with Severe and Nonsevere Invasive Diseases. Infect. Immun..

[33] Krishnan K.C., Mukundan S., Alagarsamy J., Laturnus D., Kotb M. (2016). Host Genetic Variations and Sex Differences Potentiate Predisposition, Severity, and Outcomes of Group A Streptococcus-Mediated Necrotizing Soft Tissue Infections. Infect. Immun..

[34] Chella Krishnan K., Mukundan S., Alagarsamy J., Hur J., Nookala S., Siemens N., Svensson M., Hyldegaard O., Norrby-Teglund A., Kotb M. (2016). Genetic Architecture of Group A Streptococcal Necrotizing Soft Tissue Infections in the Mouse. PLoS Pathog..

[35] Tang G., Xu Z., Goldman J.E. (2006). Synergistic Effects of the SAPK/JNK and the Proteasome Pathway on Glial Fibrillary Acidic Protein (GFAP) Accumulation in Alexander Disease. J. Biol. Chem..

[36] Bellver-Landete V., Bretheau F., Mailhot B., Vallières N., Lessard M., Janelle M.E., Vernoux N., Tremblay M.È., Fuehrmann T., Shoichet M.S. (2019). Microglia Are an Essential Component of the Neuroprotective Scar That Forms after Spinal Cord Injury. Nat. Commun..

[37] Arganda-Carreras I., Kaynig V., Rueden C., Eliceiri K.W., Schindelin J., Cardona A., Seung H.S. (2017). Trainable Weka Segmentation: A Machine Learning Tool for Microscopy Pixel Classification. Bioinformatics.

[38] Talukdar S.N., Osan J., Ryan K., Grove B., Perley D., Kumar B.D., Yang S., Dallman S., Hollingsworth L., Bailey K.L. (2023). RSV-Induced Expanded Ciliated Cells Contribute to Bronchial Wall Thickening. Virus Res..

[39] Seeger D.R., Golovko S.A., Grove B.D., Golovko M.Y. (2021). Cyclooxygenase Inhibition Attenuates Brain Angiogenesis and Independently Decreases Mouse Survival under Hypoxia. J. Neurochem..

[40] Schwendy M., Unger R.E., Bonn M., Parekh S.H. (2019). Automated Cell Segmentation in FIJI^®^ Using the DRAQ5 Nuclear Dye. BMC Bioinformatics.

[41] Lopez-Rodriguez A.B., Hennessy E., Murray C.L., Nazmi A., Delaney H.J., Healy D., Fagan S.G., Rooney M., Stewart E., Lewis A. (2021). Acute Systemic Inflammation Exacerbates Neuroinflammation in Alzheimer’s Disease: IL-1β Drives Amplified Responses in Primed Astrocytes and Neuronal Network Dysfunction. Alzheimer’s Dement..

[42] Geyer S., Jacobs M., Hsu N.J. (2019). Immunity against Bacterial Infection of the Central Nervous System: An Astrocyte Perspective. Front. Mol. Neurosci..

[43] Korzhevskii D.E., Kirik O.V. (2016). Brain Microglia and Microglial Markers. Neurosci. Behav. Physiol..

[44] Bhusal A., Nam Y., Seo D., Lee W.H., Suk K. (2022). Cathelicidin-Related Antimicrobial Peptide Negatively Regulates Bacterial Endotoxin-Induced Glial Activation. Cells.

[45] Singer B.H., Dickson R.P., Denstaedt S.J., Newstead M.W., Kim K., Falkowski N.R., Erb-Downward J.R., Schmidt T.M., Huffnagl G.B., Standiford T.J. (2018). Bacterial Dissemination to the Brain in Sepsis. Am. J. Respir. Crit. Care Med..

[46] Eiche T.P., Mohajeri M.H. (2022). Overlapping Mechanisms of Action of Brain-Active Bacteria and Bacterial Metabolites in the Pathogenesis of Common Brain Diseases. Nutrients.

[47] Isaiah S., Loots D.T., Solomons R., van der Kuip M., Tutu Van Furth A.M., Mason S. (2020). Overview of Brain-to-Gut Axis Exposed to Chronic CNS Bacterial Infection(s) and a Predictive Urinary Metabolic Profile of a Brain Infected by Mycobacterium Tuberculosis. Front. Neurosci..

[48] Bottasso O., Bay M.L., Besedovsky H., del Rey A. (2013). Adverse Neuro-Immune-Endocrine Interactions in Patients with Active Tuberculosis. Mol. Cell. Neurosci..

[49] Zager A., Andersen M.L., Lima M.M.S., Reksidler A.B., Machado R.B., Tufik S. (2009). Modulation of Sickness Behavior by Sleep: The Role of Neurochemical and Neuroinflammatory Pathways in Mice. Eur. Neuropsychopharmacol..

[50] Liu Y.H., Wu P.H., Kang C.C., Tsai Y.S., Chou C.K., Liang C.T., Wu J.J., Tsai P.J. (2019). Group A Streptococcus Subcutaneous Infection-Induced Central Nervous System Inflammation Is Attenuated by Blocking Peripheral TNF. Front. Microbiol..

[51] Pillai A., Thomas S., Williams C. (2005). Clindamycin in the Treatment of Group G β-Haemolytic Streptococcal Infections. J. Infect..

[52] Andreoni F., Zürcher C., Tarnutzer A., Schilcher K., Neff A., Keller N., Marques Maggio E., Poyart C., Schuepbach R.A., Zinkernagel A.S. (2017). Clindamycin Affects Group a Streptococcus Virulence Factors and Improves Clinical Outcome. J. Infect. Dis..

[53] Cunningham M.W., Cox C.J. (2016). Autoimmunity against Dopamine Receptors in Neuropsychiatric and Movement Disorders: A Review of Sydenham Chorea and Beyond. Acta Physiol..

[54] Korn T., Hiltensperger M. (2021). Role of IL-6 in the Commitment of T Cell Subsets. Cytokine.

[55] Laho D., Blumental S., Botteaux A., Smeesters P.R. (2021). Invasive Group A Streptococcal Infections: Benefit of Clindamycin, Intravenous Immunoglobulins and Secondary Prophylaxis. Front. Pediatr..

[56] Avire N.J., Whiley H., Ross K. (2021). A Review of Streptococcus Pyogenes: Public Health Risk Factors, Prevention and Control. Pathogens.

[57] Burmeister A.R., Marriott I. (2018). The Interleukin-10 Family of Cytokines and Their Role in the CNS. Front. Cell. Neurosci..

[58] Ottolenghi A., Bolel P., Sarkar R., Greenshpan Y., Iraqi M., Ghosh S., Bhattacharya B., Taylor Z.V., Kundu K., Radinsky O. (2021). Life-Extended Glycosylated IL-2 Promotes Treg Induction and Suppression of Autoimmunity. Sci. Rep..

[59] Harris F., Berdugo Y.A., Tree T. (2023). IL-2-Based Approaches to Treg Enhancement. Clin. Exp. Immunol..

[60] Ciccia F., Guggino G., Rizzo A., Saieva L., Peralta S., Giardina A., Cannizzaro A., Sireci G., De Leo G., Alessandro R. (2015). Type 3 Innate Lymphoid Cells Producing IL-17 and IL-22 Are Expanded in the Gut, in the Peripheral Blood, Synovial Fluid and Bone Marrow of Patients with Ankylosing Spondylitis. Ann. Rheum. Dis..

[61] Liu C., Yang J. (2022). Enteric Glial Cells in Immunological Disorders of the Gut. Front. Cell. Neurosci..

[62] Si Y., Zhang Y., Zuloaga K., Yang Q. (2023). The Role of Innate Lymphocytes in Regulating Brain and Cognitive Function. Neurobiol. Dis..

[63] Gong J., Zhan H., Liang Y., He Q., Cui D. (2021). Role of Th22 Cells in Human Viral Diseases. Front. Med..

[64] Zhang K., Chen L., Zhu C., Zhang M., Liang C. (2023). Current Knowledge of Th22 Cell and IL-22 Functions in Infectious Diseases. Pathogens.

[65] Liang S.C., Tan X.Y., Luxenberg D.P., Karim R., Dunussi-Joannopoulos K., Collins M., Fouser L.A. (2006). Interleukin (IL)-22 and IL-17 Are Coexpressed by Th17 Cells and Cooperatively Enhance Expression of Antimicrobial Peptides. J. Exp. Med..

[66] Thorsdottir S., Henriques-Normark B., Iovino F. (2019). The Role of Microglia in Bacterial Meningitis: Inflammatory Response, Experimental Models and New Neuroprotective Therapeutic Strategies. Front. Microbiol..

[67] Afridi R., Kim J.-H., Rahman M.H., Suk K. (2020). Metabolic Regulation of Glial Phenotypes: Implications in Neuron–Glia Interactions and Neurological Disorders. Front. Cell. Neurosci..

[68] Kim Y.S., Joh T.H. (2006). Microglia, Major Player in the Brain Inflammation: Their Roles in the Pathogenesis of Parkinson’s Disease. Exp. Mol. Med..

[69] Cenker J.J., Stultz R.D., McDonald D. (2017). Brain Microglial Cells Are Highly Susceptible to HIV-1 Infection and Spread. AIDS Res. Hum. Retroviruses.

[70] Garden G.A. (2002). Microglia in Human Immunodeficiency Virus-Associated Neurodegeneration. Glia.

[71] Wang T., Gong N., Liu J., Kadiu I., Kraft-Terry S.D., Mosley R.L., Volsky D.J., Ciborowski P., Gendelman H.E. (2008). Proteomic Modeling for HIV-1 Infected Microglia-Astrocyte Crosstalk. PLoS ONE.

[72] Ton H., Xiong H. (2013). Astrocyte Dysfunctions and HIV-1 Neurotoxicity. J. AIDS Clin. Res..

[73] Hamadi N., Sheikh A., Madjid N., Lubbad L., Amir N., Shehab S.A.D.S., Khelifi-Touhami F., Adem A. (2016). Increased Pro-Inflammatory Cytokines, Glial Activation and Oxidative Stress in the Hippocampus after Short-Term Bilateral Adrenalectomy. BMC Neurosci..

[74] Murray C.A., Lynch M.A. (1998). Evidence That Increased Hippocampal Expression of the Cytokine Interleukin-1β Is a Common Trigger for Age- and Stress-Induced Impairments in Long-Term Potentiation. J. Neurosci..

[75] Hosseini S., Wilk E., Michaelsen-Preusse K., Gerhauser I., Baumgärtner W., Geffers R., Schughart K., Korte M. (2018). Long-Term Neuroinflammation Induced by Influenza a Virus Infection and the Impact on Hippocampal Neuron Morphology and Function. J. Neurosci..

[76] Fu H., Hardy J., Duff K.E. (2018). Selective Vulnerability in Neurodegenerative Diseases. Nat. Neurosci..

